# Reemergence of epidemic malaria in the highlands of western Kenya.

**DOI:** 10.3201/eid0404.980422

**Published:** 1998

**Authors:** M A Malakooti, K Biomndo, G D Shanks

**Affiliations:** University of the Health Sciences, Bethesda, Maryland, USA. malakooti@pol.net

## Abstract

Hospital records (1990-1997) of a tea company in the Kericho district, western Kenya, showed malaria epidemics almost annually from May to July, with an annual attack rate of 50%, 857 hospitalizations per 100,000 per year, and 42 deaths per 100,000 per year; 32% of deaths in hospitalized patients were caused by malaria. A questionnaire survey (June 1997) of 244 patients hospitalized for malaria showed that only 8% had traveled to an area with known malaria transmission 30 days before diagnosis. The increasing malaria incidence may be due to drug resistance.


### Reemergence of Epidemic Malaria in the Highlands of Western Kenya

**M. A. Malakooti*, K. Biomndo†, and G. D. Shanks‡**
*University of the Health Sciences, Bethesda, Maryland, USA;
†Brooke Bond Central Hospital, Kericho, Kenya; ‡US Army
Medical Research Unit, Nairobi, Kenya

Hospital records (1990-1997) of a tea company in the Kericho district, western Kenya, showed malaria epidemics almost annually from May to July, with an annual attack rate of 50%, 857 hospitalizations per 100,000 per year, and 42 deaths per 100,000 per year; 32% of deaths in hospitalized patients were caused by malaria. A questionnaire survey (June 1997) of 244 patients hospitalized for malaria showed that only 8% had traveled to an area with known malaria transmission 30 days before diagnosis. The increasing malaria incidence may be due to drug resistance.

This study investigated reports of epidemic malaria in a highland area of Kenya traditionally thought to be free of malaria to 1) formulate recommendations for reducing disease incidence and preventing future epidemics and 2) determine whether malaria was imported or was due to local transmission.

The factors necessary for malaria transmission are the *Plasmodium* parasite, the *Anopheles* mosquito vector, and the human host. Both parasite and vector are affected by temperature and rainfall. Although temperature extremes kill *Anopheles* mosquitoes, higher temperatures (15°C–30°C) increase their rate of development. The gonotrophic cycle (interval between blood meals) shortens with increasing temperature, and the effect of a small temperature increase is greatest at the cooler ambient temperatures. Thus, a small rise in temperature from 19°C to 21°C shortens the gonotrophic cycle from 4 to 3 days and increases the vectorial capacity of the mosquito (1). Altitude is thought to be a proxy for temperature, so the actual limiting factor for malaria at high altitude is the effect of the lower temperature on the parasite (2). Despite the effect of altitude on ambient temperature, microclimatic factors (e.g. heated houses) can play an important role in facilitating malaria transmission and epidemics at higher elevations. Unlike the parasite, the mosquito vector can commonly be found at altitudes from >1,600 m (3,4) to 3,000 m, demonstrating that the limiting factor for malaria transmission at high altitude is the survival of the *Plasmodium* parasite. The *Anopheles* population is very sensitive to rainfall, which increases the availability of mosquito breeding sites. *A. gambiae,* the primary malaria vector of western Kenya, lays its eggs in small pools and puddles; in this region, rainfall of 150 mm per month leads to rapid expansion of the *A. gambiae* population (3,5) and hence increased risk for malaria transmission. Finally, epidemic malaria requires sufficient numbers of human gametocyte carriers (to infect *Anopheles* mosquitoes) and nonimmune human hosts (to acquire clinical infection).

Malaria did not exist in the western Kenya highlands until the second decade of the 20th century (6). In 1901, completion of a railway from the Kenya coast across the highlands and down to Lake Victoria and increased road transport facilitated the gradual spread of infective mosquitoes into the highlands from the low-lying hyperendemic-disease areas (5). The development of tea estates and agriculture in the highlands, with the concomitant clearing of the forests, provided suitable mosquito breeding grounds. Finally, importation of infected laborers completed the conditions necessary for malaria transmission.

Address for correspondence: M. A. Malakooti, MD, MTM&H, Epidemiology Department, Navy Environmental and Preventive Medicine Unit #2, 1887 Powhatan Street, Norfolk, VA 23511-3394, USA; fax: (757) 444-1191; e-mail: malakooti@pol.net.

The first reported epidemic was in 1918 to 1919 when local soldiers returned from Tanzania after World War I (6). Two epidemics were recorded in the 1920s and four in the 1930s. Garnham (2) reported epidemics of malaria in the Londiani area of western Kenya from 1941 to 1944, close to the site of the present study, at an altitude of approximately 2,200 m. After the military camp in the area was disbanded in 1944, the local outbreaks ceased, but highland malaria continued to be a serious public health problem until the late 1950s, when an extensive control program essentially eliminated the disease (7). The highlands were considered free of malaria through the 1960s, but since the 1980s malaria has been increasing (8).

More than 90% of malaria in Kenya is caused by *P. falciparum* (4,5) and transmitted most often by *A. gambiae*, with *A. funestus* as a secondary vector. It has always been assumed that malaria in the highlands of western Kenya is not due to local transmission but is imported from the nearby holoendemic-disease areas around Lake Victoria by the frequent travel of the tea plantation workers and their families.

### The Study

### Geography

This study was conducted in the Kericho tea-growing area at 0° 22' south and 35° 17' east in the Rift Valley highlands of western Kenya, 80 km from the holoendemic malaria area of the Lake Victoria basin. The altitude of the tea estates is 1,780 m to 2,225 m; the mean monthly temperature is 18.7°C. February is the warmest month of the year (Figure 1), with a mean maximum daily temperature of 28.3°C and mean minimum daily temperature of 10.8°C; July is the coolest month, with a mean maximum daily temperature of 25.4°C and mean minimum daily temperature of 10.2°C. Annual rainfall is 1.79 m with a March-to-June rainy season, and relative humidity is generally slightly below 60% except for May to July. April is the wettest month, with mean rainfall of 252 mm; December is the driest with a mean rainfall of 78 mm.

### Demographics

The study data were obtained from the health-care system of a major tea company, which has 18 estates with 22,000 workers and approximately 100,000 persons eligible for health care; 32% of company employees belong to ethnic groups whose traditional home areas have holo/hyperendemic malaria. Given that marriage between ethnic groups is not common, it follows that about 30,000 workers and dependents are from malarious areas; most people travel back to their home regions at least once per year. Approximately 31% of employees originate from the highland areas and the remaining 37% from nonendemic-disease areas around the country. This labor composition has remained stable for at least 20 to 30 years. The employees and families generally live in cement-walled, corrugated metal-roofed duplex style houses, situated close together in large housing areas. The houses usually have 1½ rooms, with a wood or charcoal-burning fireplace, one door, and two unscreened windows. The Central Hospital has 67 beds, two physicians, and two clinical officers, and averages 50 to 80 hospitalized patients per day and 20,000 outpatients per year. Three outlying medical centers, each with

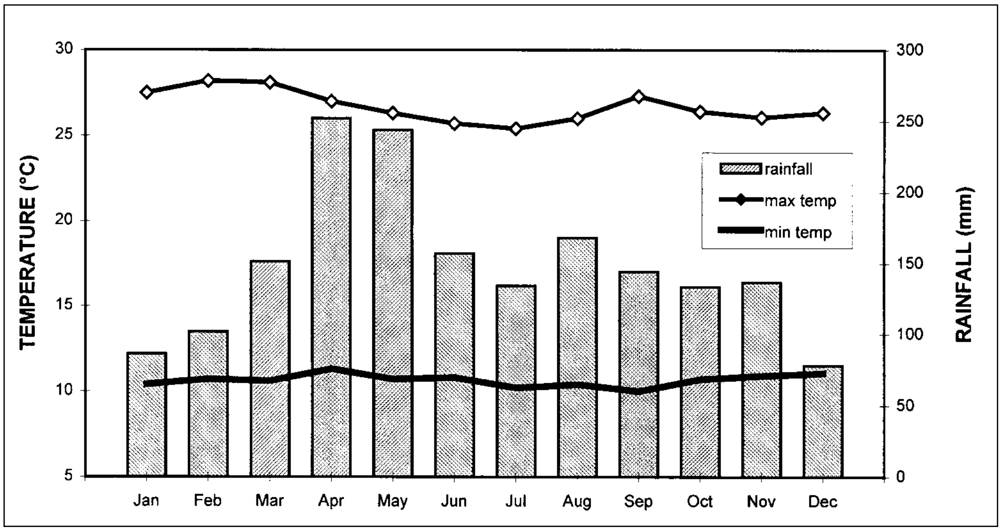

Figure 1. Mean monthly rainfall (1971–97), maximum and minimum temperatures (1984–97), Kericho area.

a clinical officer, see 15,000 to 20,000 patients per year; and 26 dispensaries, each with a nurse provider, see approximately 180,000 to 240,000 outpatients per year.

### Data Sources

Retrospective study data were extracted from the Central Hospital monthly reports, which include inpatient and outpatient illness, death, and laboratory data for the whole health-care system. To determine local risk factors for malaria infection, we used daily logbooks on the inpatient wards to identify patients admitted with malaria during June 1997; a simple questionnaire was field tested in consultation with hospital staff and administered to 244 of the 257 identified malaria patients hospitalized during June 1997. The questionnaire asked for demographic and recent travel history and was administered by the primary investigator with a local field worker/translator or by one of the hospital clinical officers.

### Findings

Central Hospital illness and death data were available from 1990 through 1997, except for outpatient and death data from October to December 1992. Tea company temperature data were available from 1984 through 1997, and rainfall data from 1971 through 1997 (Figures 1 and 2). The retrospective illness data clearly show the nearly annual occurrence of epidemic malaria (Figures 2 and 3) and the importance of known factors in highland malaria transmission; rainfall >150 mm per month in this area promotes epidemic malaria, and average temperature <18°C appears to extinguish epidemics. In the Kericho area, malaria epidemics occur during May to July, after the long rains and before the temperature drops in July (Figure 3). Figure 4 shows outpatient and inpatient malaria and malaria deaths from 1991 through 1997. For outpatient malaria, the mean annual rate was 48,112 per 100,000 (range 33,914 to 56,040 per 100,000), i.e., each year malaria was diagnosed in approximately 50% of company health-care beneficiaries; for malaria admissions the mean annual rate was 857 per 100,000 (range 361 to 1,058 per 100,000); and for malaria deaths the mean annual rate was 42 per 100,000 (range 14 to 64 per 100,000). In hospitalized patients malaria diagnosis was laboratory confirmed, but in most outpatients malaria was diagnosed purely on a clinical basis. Of the 764 inpatient deaths documented from 1991 through 1997, 254 (32%) were caused by malaria, the leading cause of death in this highland population. Of the 254 malaria deaths, 37% were recorded as being due to severe anemia, 31% cerebral malaria, and 32% malaria with gastroenteritis or pneumonia.

Questionnaire results were available for 244 (95%) of the 257 patients admitted for malaria in June 1997. The remaining 13 patients were either discharged before interview and could not be traced to their houses on the estates, died before interview and the family was not available for questioning, or were minors whose parents were not available for interview. These malaria

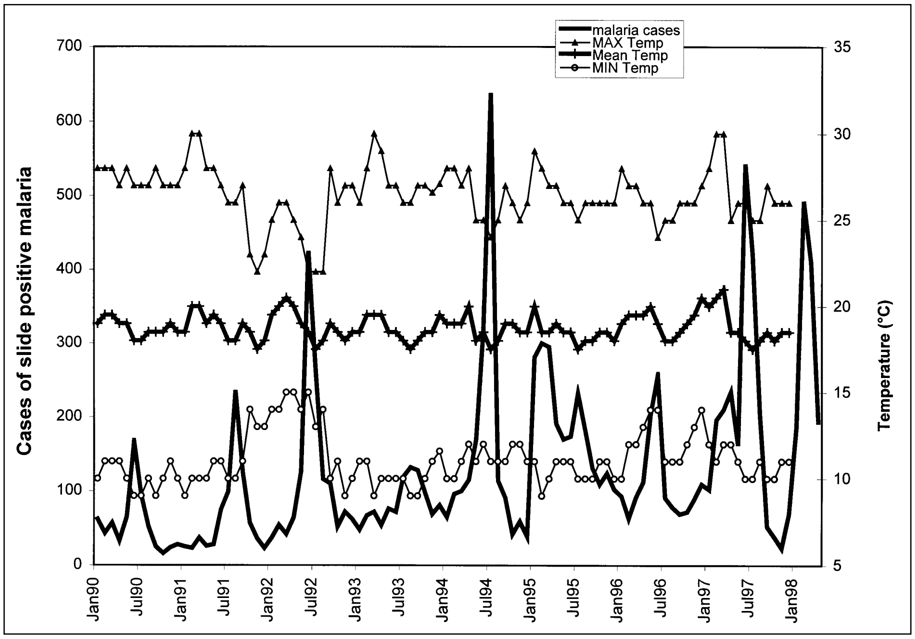

Figure 2. Monthly cases of slide-positive malaria versus maximum, minimum, and mean temperature.

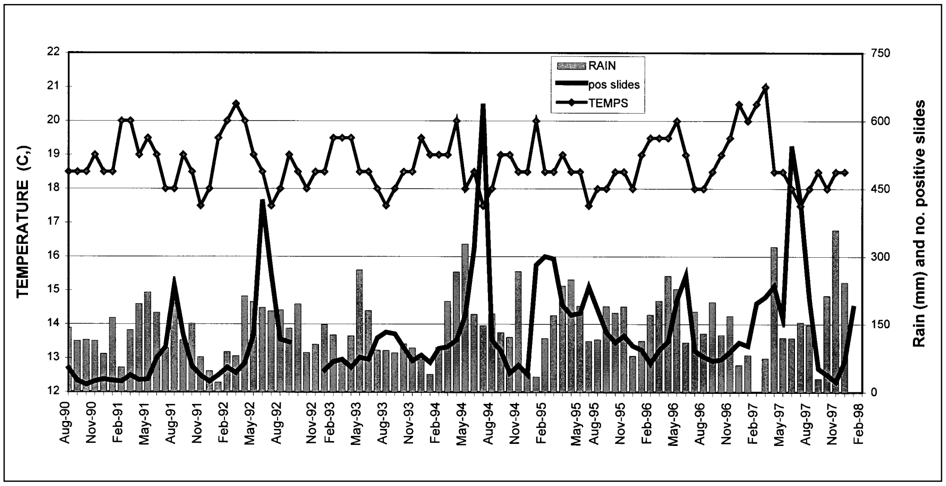

Figure 3. Positive slides, rainfall, and average temperature, per month.

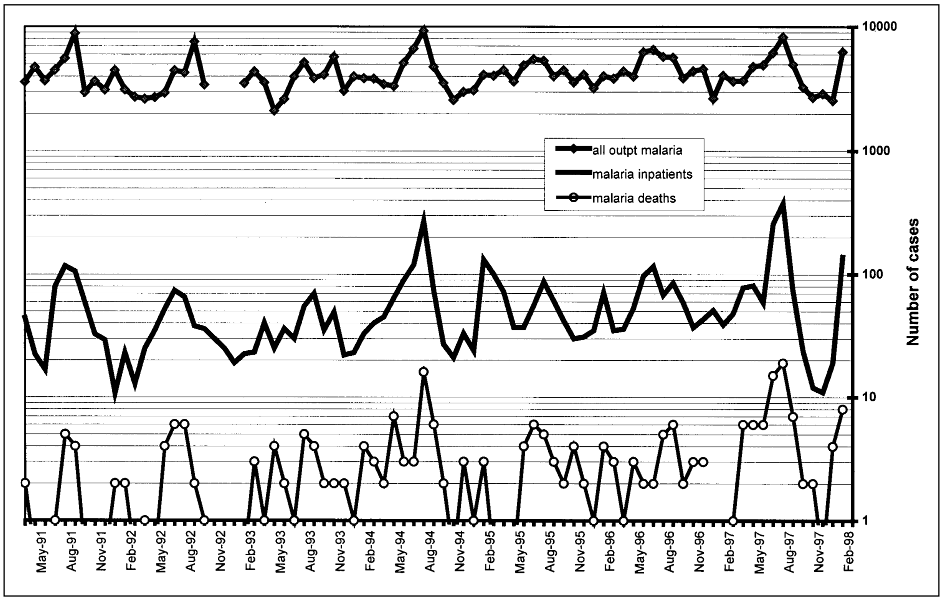

Figure 4. Malaria outpatients, inpatients, and deaths, versus time.

hospitalized patients were 48% female, 52% male, with mean age 13.2 years (range 1 month to 58 years); 20% were tea company employees; 80% were dependents. Only 16% of malaria hospitalized patients belonged to ethnic groups originating from areas with holo/hyperendemic malaria, versus 32% of all company employees (relative risk = 0.4 [95% confidence interval 0.29 to 0.57] for hospitalization for malaria if ethnic origin was from a holoendemic-disease area versus a nonendemic disease area). Forty-three (18%) of the patients hospitalized with malaria had traveled away from the Kericho area in the 30 days before admission, with an average of 19 days away (range 1 to 90 days). Of the 43 hospitalized patients who had traveled, 19 had been to malarious areas; therefore, only 8% of the 244 malaria hospitalized patients had recently traveled to an area with known malaria transmission.

### Conclusions

These data document the reemergence of recurrent epidemic malaria due to local transmission in the highlands of western Kenya. Although malaria in the highlands was a serious problem after World War II, because of improved transportation and increased immunity, recurrent epidemic malaria had not been seen in a generation. Several pertinent factors have remained stable and do not appear to explain the increase in malaria in this area. Firstly, climate data from the Kericho area show no obvious change in average temperature or rainfall over the last 10 to 20 years that would explain the present almost annual epidemics in the highlands. Although the climate data used in this study may not be sensitive enough to detect small changes in environmental temperature, global warming would also not explain the epidemics seen in the 1940s to 1950s. Deforestation may be relevant, providing more breeding sites for vector mosquitoes in sunlit pools and perhaps leading to localized changes in weather patterns and increased microenvironmental temperatures (1).

Secondly, travel of infected persons from adjacent malaria-endemic areas is not new. The main road from the malaria-holoendemic Lake Victoria basin to this highland area was built in the 1950s; in fact, people had been making the same journey using ox wagons decades before the road was paved (5,6). According to the tea company's central office, the tribal/ethnic composition of the employees has remained stable at least since the 1970s, as has the total number of health-care system beneficiaries. Therefore, the increase in malaria does not seem to be explained by an increase in the number of nonimmune relative to semiimmune/immune persons nor simply by an increase in the total population.

A third factor that could lead to an impression of increasing malaria would be the degradation of the health-care infrastructure: if the system became overwhelmed by other diseases and lack of funding, it would be unable to cope with the same number of malaria cases as before, leading to increased hospitalizations and deaths. Indeed, the public health sector in much of sub-Saharan Africa has been overwhelmed by the AIDS epidemic, lack of funding, and other institutional problems; however, the tea company in this study continues to provide excellent care.

One hypothesis for the increase in highland malaria is the failure of the drugs used to cure malaria infections. The period during which epidemic malaria was absent from the highlands corresponds to the time when pyrimethamine and chloroquine were still effective malaria treatments. In 1953, pyrimethamine was used successfully as the first step of a large malaria control campaign in the Kenya highlands, but by 1954, rapid development of resistance was reported in several areas (9,10). Nevertheless, widespread use of chloroquine as well as incorrect use of other malaria medications continue. Clinical control of symptoms (but not cure) creates a large number of semiimmune gametocyte carriers, who can infect mosquitoes when transmission is favored at midyear. In this way, the failure to cure falciparum malaria infections leads to an increase in the human gametocyte-carrier pool, resulting in the rapid spread of epidemic malaria among the largely nonimmune highland population. Studies are under way to further investigate this hypothesis. While increased drug resistance may be the major factor in the documented increase of epidemic malaria, the causes are undoubtedly multifactorial and include vector, host, and environmental components.

Several possible interventions to stop the cycle of epidemic malaria and the associated high costs may be worth exploring. Spraying of houses on the tea estates with residual insecticide could be effective against the endophilic and endophagic *A. gambiae* (11), which appears to be the primary vector in these highlands (5,7). Indoor spraying campaigns in the past in western Kenya with DDT (12) and Dieldrin (7) have effectively combated malaria epidemics. Even now, worker acceptability would likely not be an issue as houses are regularly sprayed for other public health reasons. Insecticide-impregnated bed nets may also be effective with proper education and follow-up. Finally, improved treatment regimens to cure malaria infections, gametocytocidal drugs to prevent transmission, and chemoprophylaxis of workers during travel to malaria-holoendemic areas should be evaluated. Protection of a well-defined, economically important, and less immune population may make effective certain strategies of drug use, such as intermittent chemoprophylaxis, which would not normally be considered in areas with higher levels of malaria endemicity.

Dr. Malakooti is a preventive medicine physician, Epidemiology Department, Navy Environmental and Preventive Medicine Unit No. 2, Norfolk, Virginia, USA. His main interests are in tropical medicine and international health, particularly as related to malaria.

### Acknowledgments

We thank the Management of Brooke Bond Kenya Ltd.; Florence Chepkwony and the staff of Brooke Bond Central Hospital; Hussein Sijenje; Aggrey Oloo of the Kenya Medical Research Institute; and Robert Snow and other colleagues who have commented on the manuscript. This paper is published with permission of the director, Kenya Medical Research Institute.

### References

1. Lindsay SW, Birley MH. Climate change and malaria transmission. Ann Trop Med Parasitol 1996;90:573-88.
2. Garnham PCC. Malaria epidemics at exceptionally high altitudes in Kenya. BMJ 1945;2:45-7.

3. Githeko AK, Service MW, Nbongo CM, Atieli FK. Resting behaviour, ecology and genetics of malaria vectors in large scale agricultural areas of Western Kenya. Parassitologia 1996;38:481-9.
4. Khaemba BM, Mutani A, Bett MK. Studies of Anopheline mosquitoes transmitting malaria in a newly developed highland urban area: a case study of Moi University and its environs. East Afr Med J 1994;71:159-64.
5. Garnham PCC. The incidence of malaria at high altitudes. Journal of the National Malaria Society 1948;7:275-84.
6. Matson AT. The history of malaria in Nandi. East Afr Med J 1957;34:431-41.
7. Roberts JMD. The control of epidemic malaria in the highlands of western Kenya, Part III. After the campaign. J Trop Med Hyg 1964;59:230-7.
8. Oloo AJ, Vulule JM, Koech DK. Some emerging issues on the malaria problem in Kenya. East Afr Med J 1996;70:50-3.
9. Clyde DF. Observations on monthly pyrimethamine ("Daraprim") prophylaxis in an East African village. East Afr Med J 1954;31:41-6.
10. Avery Jones S. Resistance of *P. falciparum* and *P. malariae* to pyrimethamine (Daraprim) following mass treatment with this drug. East Afr Med J 1954;31:47-9.
11. Gilles HM, Warrell DA, editors. In: Bruce-Chwatt's Essential Malariology. 3rd ed. London: Edward Arnold; 1993.
12. Roberts JMD. The control of epidemic malaria in the highlands of western Kenya, Part I. Before the campaign. J Trop Med Hyg 1964;59:161-71.
